# Chitosan/Glycosaminoglycan Scaffolds: The Role of Silver Nanoparticles to Control Microbial Infections in Wound Healing

**DOI:** 10.3390/polym11071207

**Published:** 2019-07-19

**Authors:** Giuseppina Sandri, Dalila Miele, Angela Faccendini, Maria Cristina Bonferoni, Silvia Rossi, Pietro Grisoli, Angelo Taglietti, Marco Ruggeri, Giovanna Bruni, Barbara Vigani, Franca Ferrari

**Affiliations:** 1 Department of Drug Sciences, University of Pavia, viale Taramelli 12, 27100 Pavia, Italy; 2 Department of Chemistry, University of Pavia, viale Taramelli 12, 27100 Pavia, Italy

**Keywords:** chitosan, chondroitin sulfate, hyaluronic acid, silver nanoparticles, electrospun skin scaffold, antimicrobial properties, enzymatic degradation

## Abstract

Cutaneous wounds represent a major issue in medical care, with approximately 300 million chronic and 100 million traumatic wound patients worldwide, and microbial infections slow the healing process. The aim of this work was to develop electrospun scaffolds loaded with silver nanoparticles (AgNPs) to enhance cutaneous healing, preventing wound infections. AgNPs were directly added to polymeric blends based on chitosan (CH) and pullulan (PUL) with hyaluronic acid (HA) or chondroitin sulfate (CS) to be electrospun obtaining nanofibrous scaffolds. Moreover, a scaffold based on CH and PUL and loaded with AgNPs was prepared as a comparison. The scaffolds were characterized by chemico–physical properties, enzymatic degradation, biocompatibility, and antimicrobial properties. All the scaffolds were based on nanofibers (diameters about 500 nm) and the presence of AgNPs was evidenced by TEM and did not modify their morphology. The scaffold degradation was proven by means of lysozyme. Moreover, the AgNPs loaded scaffolds were characterized by a good propensity to promote fibroblast proliferation, avoiding the toxic effect of silver. Furthermore, scaffolds preserved AgNP antimicrobial properties, although silver was entrapped into nanofibers. Chitosan/chondroitin sulfate scaffold loaded with AgNPs demonstrated promotion of fibroblast proliferation and to possess antimicrobial properties, thus representing an interesting tool for the treatment of chronic wounds.

## 1. Introduction

Chronic, non-healing wounds (often termed ulcers) can be defined as wounds with a full thickness in depth and a slow healing tendency (higher than 12 weeks), thus failing to proceed through an orderly and timely reparative process. This causes an inability to produce skin having anatomic and functional integrity and the repair process proceeds without the restoring of anatomic and functional skin integrity [1]. Based on the causative aetiologies, the Wound Healing Society classifies chronic wounds into four categories: pressure ulcers, diabetic ulcers, venous ulcers, and arterial insufficiency ulcers [1]. Chronic wounds are often disguised as a comorbid condition and represent a silent epidemy that affects a large fraction of the world population. A rough prevalence of chronic non-healing wounds in developed countries is 1% to 2% of the general population, similar to the prevalence rate for heart failure. However, unlike hearth diseases, the morbidity and the costs associated have been largely ignored, probably due to no specific medical specialty, which is clearly responsible for this [2]. However, non-healing wounds are estimated as increasing morbidity, mainly due to the dramatic ageing of the population, as wound healing is negatively associated with age [1]. Complications of chronic wounds include infections (an open wound is a favorable niche for microbial colonization) as cellulitis and infective venous eczema, gangrene, and haemorrhage that could lead to lower-extremity amputations and ultimately to a disability.

Generally, infections are responsible for postponing wound healing and the majority of infected wounds present polymicrobial contamination with pathogen strains normally founded in the surrounding environment. There is no consensus about the opportunity for treating chronic wounds with chemotherapeutics/antibiotics to prevent infections, since they could trigger bacteria resistance. For this reason, antibacterials could circumvent the problem [3].

Silver has been used as antimicrobial agent from centuries [4]. Moreover, silver nanoparticles (AgNPs) have gained considerable attention due to their broad inhibitory activity towards various strains of bacteria and more importantly against antibiotic resistant bacteria [5]. Despite various hypotheses being available, the mechanisms of the antibacterial properties of AgNPs so far have not been established clearly [6] and there are several hypotheses to explain their activity.

In particular AgNPs seem to adhere onto the membrane surface of microbial cells, modifying the lipid bilayer and increasing the membrane permeability, moreover Ag^+^ ions released by means of oxidation of silver seem to penetrate into the cells and to specifically interact with respiratory chain enzymes, nucleic acids, and/or cytoplasmic components, thus modulating intracellular signal transduction pathways. Moreover, silver oxidation could generate ROS (Reactive Oxygen Species) and radicals able to damage intracellular micro-organelles (i.e., mitochondria, ribosomes, and vacuoles) and biomolecules including DNA, protein, and lipids [6]. However, AgNPs do not only show broad antimicrobial activity, but also broad cytotoxicity towards mammalian cells [7,8].

Given this premise, the aim of this work was the development of chitosan/glycosaminoglycan electrospun scaffolds loaded with AgNPs to control microbial infections in wound healing. Chitosan (CH) was blended with chondroitin sulfate (CS) or hyaluronic acid (HA) and pullulan (P) was added to both the blends to have CH/CS and CH/HA scaffolds. A scaffold based on P and CH without glycosaminoglycans was used as a comparison (CH scaffolds). Preliminary enzymatic degradation of the systems was assessed using lysozyme, normally secreted by white cells (macrophages and neutrophilis during inflammatory phase): this was the starting point for the assessment of their employment as dermal substitutes. Subsequently, the scaffolds were loaded with AgNPs, as antimicrobial agent to avoid scaffold contamination and colonization during the healing process. Then, the scaffolds were characterized for chemico–physical properties (morphology—SEM, and AgNP entrapment—TEM, and structure—FTIR) and for biopharmaceutical properties (in vitro biocompatibility towards fibroblasts, cell adhesion and proliferation, and antimicrobial properties against *E. coli* and *S. aureus*).

## 2. Materials and Methods

### 2.1. Materials

For silver nanoparticles (AgNPs) synthesis the following reagents were used: sodium borohydride (98%), l-ascorbic acid (AA) (≥99%), silver nitrate (99.8%), and sodium citrate (≥99%) (Sigma Aldrich, Milan, Italy).

For the scaffold preparation the following polysaccharides were used: Chitosan (CH) (β-(1-4)-linked d-glucosamine and *N*-acetyl-d-glucosamine) low *M*_w_ 251 kDa, deacetylation degree 98%, (ChitoClear, Siiiglufjordur, Iceland); chondroitin sodium sulfate (CS) (β-1,4-linked d-glucuronic acid and β-1,3-linked *N*-acetyl galactosamine) bovine 100 EP, low *M*_w_ 14 kDa, mixture of chondroitin A (chondroitin 4 sulfate) and chondroitin C (chondroitin 6 sulfate) (Bioiberica, Barenz, Italy); hyaluronic acid (HA) (based on β-1,3-linked *N*-acetylglucosamine and β-(1,4)-d-glucuronic acid) low *M*_w_ 212 kDa (Bioiberica, Barenz, Italy); pullulan (P) (based on maltotriose repeating units, linear α 1-4 and α 1-6 glucan, produced by *Aureobasidium pullulans*) low *M*_w_ ~200–300 kDa (food grade, Hayashibara, Japan, Giusto Faravelli, Milan, Italy).

Citric acid (CA) (monohydrated citric acid, EP grade, Carlo Erba, Milan, Italy) was used as a crosslinking agent.

Ultrapure water was obtained from a Milli Q^®^ apparatus (Millipore^®^, Milan, Italy).

### 2.2. Methods

#### 2.2.1. Unloaded Nanofibrous Scaffolds Preparation

The nanofibrous scaffolds were previously developed and characterized [9]. Briefly, three different polymeric blends were prepared by mixing P, P/CS, and P/HA with CH solution at 1:1 weight ratio. CH solution was prepared in 90% *v*/*v* acetic acid and CA added and blended with P solution in water for CH scaffold or P and CS for CH/CS scaffold or P and HA for CH/HA scaffold. The blend composition is reported in Table 1.

Scaffolds were prepared using CH, CH/CS, and CH/HA blends by means of an electrospinning apparatus (STKIT-40, Linari Engineering, Pisa, Italy), equipped with a high-voltage power supply (Razel R99-E 40, Razel Scientific, Saint Albans, VT, USA), a 10 mL syringe with 21 G needle (0.8 × 20 mm), and a conductive static collector, covered by an aluminum foil. Unloaded CH, CH/CS, and CH/HA scaffolds were then crosslinked by heating at 150°C for 1 h. This process has also been reported as able to dry sterilize the products [10].

#### 2.2.2. Unloaded Nanofibrous Scaffold Degradation via Lysozyme

An exact amount of each scaffold was dipped in 1 mL of phosphate buffer 0,05 M (pH 6.2) containing 3.3 mg/mL of lysozyme (120,530 U/mg, Sigma Aldrich, Milan, Italy) at 25 °C for 24 h. After 24 h the solution was withdrawn, and the scaffold was dipped in 1 mL of fresh phosphate buffer in presence of lysozyme. This procedure was followed for 10 days. In parallel, the same experiment was performed using the same procedure without lysozyme to evaluate the eventual interferences.

Each sample was subjected to glucosamine quantification (product of chitosan degradation due to lysozyme) by means of ninhydrin assay [11]. For this purpose, the samples were diluted 1:1 with 1 N perchloric acid and centrifuged at 5000 rpm for 15 min, to precipitate lysozyme in solution.

Subsequently, under nitrogen blanket ninhydrin reagent (ninhydrin 2% (*w*/*v*), hydrindantin 6.8 mg/L in 3:1 (*v*/*v*) DMSO:lithium acetate buffer 4 M, pH 5.2; Sigma–Aldrich, Milan, Italy) was added to each sample at 1:1 volume ratio. Each sample was heated at 100 °C for 8 min under stirring in a shaking bath. Subsequently the samples were vortexed for 15 s to eliminate the excess of hydrindantin by oxidation. Then the samples were cooled and diluted 1:10 (*v*/*v*) using a 1:1 ethanol:water mixture. The absorbance of each sample was assayed at 570 nm (ELISA Plate Reader, “iMARK Microplate Absorbance Reader” BioRad, Milan, Italy). A calibration curve was performed and glucosamine (concentrations: 0.1, 0.075, 0.05, 0.025, and 0.0125 mg/mL) was dissolved in phosphate buffer 0.05 M pH 6.2 at 25 °C. The calibration curve was linear in the range considered with *R*^2^ values always higher than 0.9995 [12].

The scaffolds, subjected to lysozyme activity for 10 days, were dried and analyzed by means of Scanning Electron Microscopy (SEM) to evidence the fate and the morphology of nanofibers after degradation. For this purpose, scaffolds were placed on stubs, sputtered with gold, and analyzed by means of SEM (scanning electron microscopy) (Stereoscan 440 microscope, Leica Microsystems, Bensheim, Germany). 

#### 2.2.3. AgNP Nanofibrous Scaffolds Preparation

##### Synthesis of Silver Nanoparticles (AgNPs)

AgNPs were synthesized according to a previously reported method [9]. Briefly, in an ice cooled water, 100 mL of AgNP colloidal suspension was prepared by adding, in sequence, under vigorous stirring, the following components: 1 mL of 1% (*w*/*v*) AgNO_3_ solution, 1 mL of 1% sodium citrate and, 500 μL of an ice-cooled solution 0.075% *w*/*v* in NaBH_4_ and 1% *w*/*v* in sodium citrate. Subsequently the stirring was immediately stopped, to avoid coagulation. AgNP colloidal suspension was maintained in the dark and used within 3 days from preparation. AgNPs had a mean diameter of 7 nm [13]. 

##### Electrospinning Process

Analogously to unloaded scaffolds AgNPs loaded systems were prepared starting from three different polymeric blends. P, P/CS, and P/HA blends were prepared using AgNP colloidal suspension as solvent. Each blend was mixed at 1:1 weight ratio with CH solution (90% *v*/*v* acetic acid containing CA, as solvent), prepared as described in the 2.2.1 paragraph. The blend composition is reported in Table 1. 

The following parameters were used to obtain AgNPs loaded scaffolds: ΔV (voltage) = 22 kV, needle-to-collector distance = 24.5 cm, polymeric solution flux = 1 mL/h, spinning time = 1 h; for unloaded scaffolds: ΔV (voltage) = 21 kV (CH) or 15 kV (CH/CS and CH/HA), needle-to-collector distance = 20 cm (CH) or 15 cm (CH/CS and CH/HA), polymeric solution flux = 0.4 mL/h, spinning time = 1 h. AgNPs loaded CH, CH/CS and CH/HA scaffolds were then crosslinked by heating at 150 °C for 1 h. This process has also been reported as able to dry sterilize the products [10].

The Ag% on dry scaffolds was of 0.04% *w*/*w*.

#### 2.2.4. AgNP Nanofibrous Scaffolds Characterizations

##### Chemico-Physical Characterization

Scaffold morphology was analyzed by means of SEM (Stereoscan 440 microscope, Leica Microsystems, Bensheim, Germany) after gold sputtering. The scaffolds were analyzed before and after the crosslinking procedure and after 1 week of hydration in distilled water. Nanofiber diameters were determined by an image analysis software (Image J, ICY, Institute Pasteur, Paris, France).

AgNPs loaded in the scaffolds were analyzed by transmission electron microscopy TEM (Transmission Electron Microscopy) (Jeol JEM-1200 EX II, Tokyo, Japan) equipped with TEM CCD camera Mega View III (Jeol, Tokyo, Japan). Nanofibers were directly electrospun onto copper grids.

FT-IR (Fourier Transform InfraRed) analysis was carried out by means of FTIR BX spectrum (Perkin Elmer, Italy). The infrared spectra were acquired in the range 4000–400 cm^−1^ and unloaded scaffolds and AgNPs loaded scaffolds were compared.

##### In Vitro Cells Adhesion and Proliferation Assay

Adhesion and proliferation assays were carried out using fibroblasts (normal human dermal fibroblasts (NHDF) from juvenile foreskin, PromoCell, WVR, Milan, Italy) [14,15,16]. Fibroblasts (passages 2-5) were cultured in presence of Dulbecco’s modified Eagle medium (DMEM, Sigma-Aldrich, Italy) and supplemented with 10% fetal calf serum (FCS, Euroclone, Milan, Italy), with 200 IU/mL penicillin/0.2 mg/mL streptomycin (Sigma-Aldrich, Milan, Italy), and kept at 37 °C in a 5% CO_2_ atmosphere with 95% relative humidity (RH).

0.36 cm^2^ (0.7 cm diameter) portions of each scaffold were obtained and placed in the bottom of a well of a 96 well-plate. Fibroblasts were seeded onto each portion at 10^5^ cells/cm^2^ seeding density. The cell substrates onto the scaffolds were grown for 3 and 6 days. Fibroblasts seeded in the wells without scaffolds and grown in standard conditions were considered as control (GM). MTT (3-[4,5-dimethylthiazole-2-yl]-2,5-diphenyltetrazolium bromide) assay, SEM and CLSM (Confocal Laser Scanning Microscopy) analysis were performed as hereafter described, after 3 or 6 days of growth.

AgNP colloidal suspension containing the same silver amount as the scaffolds was compared.

MTT Test

After cell growth for 3 or 6 days, the medium was removed and 50 µL of MTT solution (Sigma Aldrich, Italy) at 2.5 mg/mL concentration in HBSS (Hank’s Buffered Salt Solution) was added with 3 h of contact time. Subsequently MTT solution was removed from each well, and the substrates were washed with 200 µL of PBS. After PBS removal, 100 µL of DMSO (dimethylsufoxide) was put in each well, and the absorbance was assayed at 570 nm by means of an ELISA plate reader (Imark Absorbance Reader, Biorad, Milan, Italy), with a reference wavelength of 690 nm. Cell viability was expressed as optical density (OD).

SEM Analysis

Cell substrates grown onto scaffolds were fixed using a 3% (*w*/*v*) glutaraldehyde solution in PBS (Sigma-Aldrich, Italy) for 2 h at room temperature. The substrates were than washed three times with PBS (Phosphate Buffered Saline) and dehydrated by washing with solutions with increasing ethanol concentration (50–75–100% *v*/*v*). Scaffolds were sputtered with gold and analyzed, as described in Section 2.2.4).

CLSM Analysis

Cells grown on the scaffolds were fixed using a 3% (*w*/*v*) glutaraldehyde solution in PBS (Sigma-Aldrich, Milan, Italy) for 2 h at room temperature. The substrates were than washed three times with PBS. Cell nuclei were stained with 100 µL of Hoechst 33258, diluted 1:10000 (Sigma-Aldrich, Italy) (10 min of contact time in the dark). Scaffolds were placed on a microscope slide and imaged using a Confocal Laser Scanning Microscope (CLSM) (Leica TCS SP2, Leica Microsystems, Wetzlar, Germany) with λ_ex_ = 346 nm and λ_em_ = 460 nm. The acquired images were processed by means of the software associated with the microscope (Leica Microsystem, Italy).

##### In Vitro Antimicrobial Assay

The antimicrobial activity of AgNPs loaded scaffolds was evaluated against two bacteria strains: *Staphylococcus aureus* ATCC 6538 and *Escherichia coli* ATCC 10356. In particular, killing time was determined as the exposure time required to kill a standardized microbial inoculum [17]. Bacteria used for killing time evaluation were grown overnight in Tryptone Soya Broth (Oxoid; Basingstoke, UK) at 37 °C. The bacteria cultures were centrifuged at 2000 rpm for 20 min to separate cells from broth and then suspended in phosphate buffer saline (PBS, pH 7.3). The suspension was diluted to adjust the number of cells to 1 × 10^7^–1 × 10^8^ CFU/mL.

A portion of each scaffold to obtain a final Ag concentration equal to 385 μg/mL was added to the microorganism suspensions.

For each microorganism, a suspension was prepared in PBS without scaffolds and used as control. Unloaded scaffolds were also tested for comparison. Bacterial suspensions were incubated at 37 °C. Viable microbial counts were evaluated after contact for 0, 5, and 24 h with scaffolds and in control suspensions; bacterial colonies were enumerated in Tryptone Soya Agar (Oxoid, Basingstoke, UK) after incubation at 37 °C for 24 h.

The microbiocidal effect (ME value) was calculated for each test organisms and contact times according to the following equation [18]:

ME = log *N*_c_ − log *N*_d_ (1)

where *N*_c_ is the number of CFU of the control microbial suspension and *N*_d_ is the number of CFU of the microbial suspension in presence of the scaffold.

##### Statistical Analysis

Statistical differences were evaluated by means of a non-parametric test: Mann Whitney (Wilcolxon) W test, (Statgraphics Centurion XV, Statistical Graphics Corporation, The Plains, VA, USA). Differences were considered significant at *p* < 0.05.

## 3. Results and Discussion

### 3.1. Nanofibrous Scaffolds Degradation Via Lysozyme

**Figure 1:**
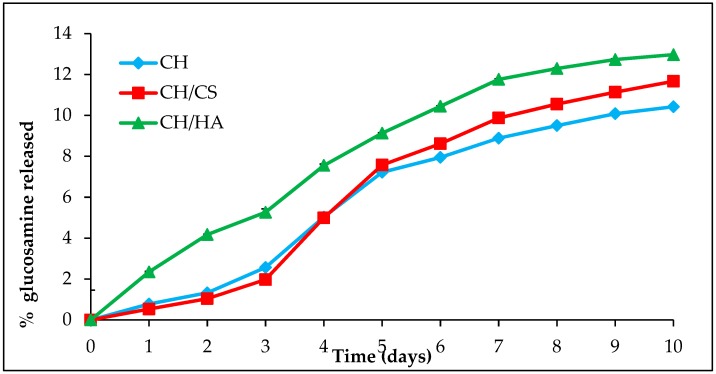
% of glucosamine released from unloaded scaffolds vs. time evaluated in the presence of lysozyme for 10 days (mean values ± sd; n = 3).

Figure 1 reports the % of glucosamine released (with respect to the theoretic amount present in the scaffolds) from unloaded scaffolds vs. time due to lysozyme activity for 10 days. The assay was performed to argue the fate of the scaffolds upon application in vivo. In fact, in a previous work [9], after 18 days of scaffold application in vivo in a murine burn excisional model, no residue was found in the histology evaluation. 

CH/HA scaffold was characterized by the highest degradation profile, while CH and CH/CS showed almost superimposable profiles and only after 6 days, CH/CS scaffold seemed to be degraded to a much greater extent than CH scaffold. This could be related to the structure of the nanofibers. In fact, in a previous work, the possible chemical modifications occurring upon crosslinking process were investigated by means of FTIR analysis performed on unloaded scaffolds before and after crosslinking. This allowed obtaining information about a fiber local response to crosslinking. This analysis highlighted that CH scaffold presented covalent bonds occurring between chitosan aminogroups and carboxylic moieties of citric acid, while CH/CS and CH/HA scaffolds were devoid of this, suggesting that the presence of anionic groups from CS (sulfate) and HA (carboxylic). These could tune covalent bond formation between CH and citric acid by means of polymer steric hindrance. Furthermore, the structural characterization at the mesoscale showed different behavior dependently of the hydration state. At the dry state, all the scaffolds were very similar and characterized by smooth fiber surface. On the contrary wet scaffolds presented hairy surface conceivably due to polymer chains protruding and stretching out from the fibers surface. Upon storage and aging in wet conditions, although no scaffold solubilization could be detected, CH/HA and CH/CS scaffolds presented the greater alterations and in particular CH/HA presented ~10 monomers polymeric chains protruding from the main structure, assuming a coiled conformation while CH/CS scaffold was characterized by a more pronounced surface roughening of the fibers that could be probably related to CS chain leakage maybe due to CS (having low molecular weight). These could explain the highest glucosamine profiles when glycosaminoglycans were present in the fibers. 

Due to the low content of AgNPs in the scaffolds, as proved also by TEM images, a negligible negative effect could be expected on the in vivo enzymatic activity, since lysozyme is continuously secreted by white cells (macrophages and neutrophilis), and in a huge amount during the inflammatory phase of wound healing.

**Figure 2:**
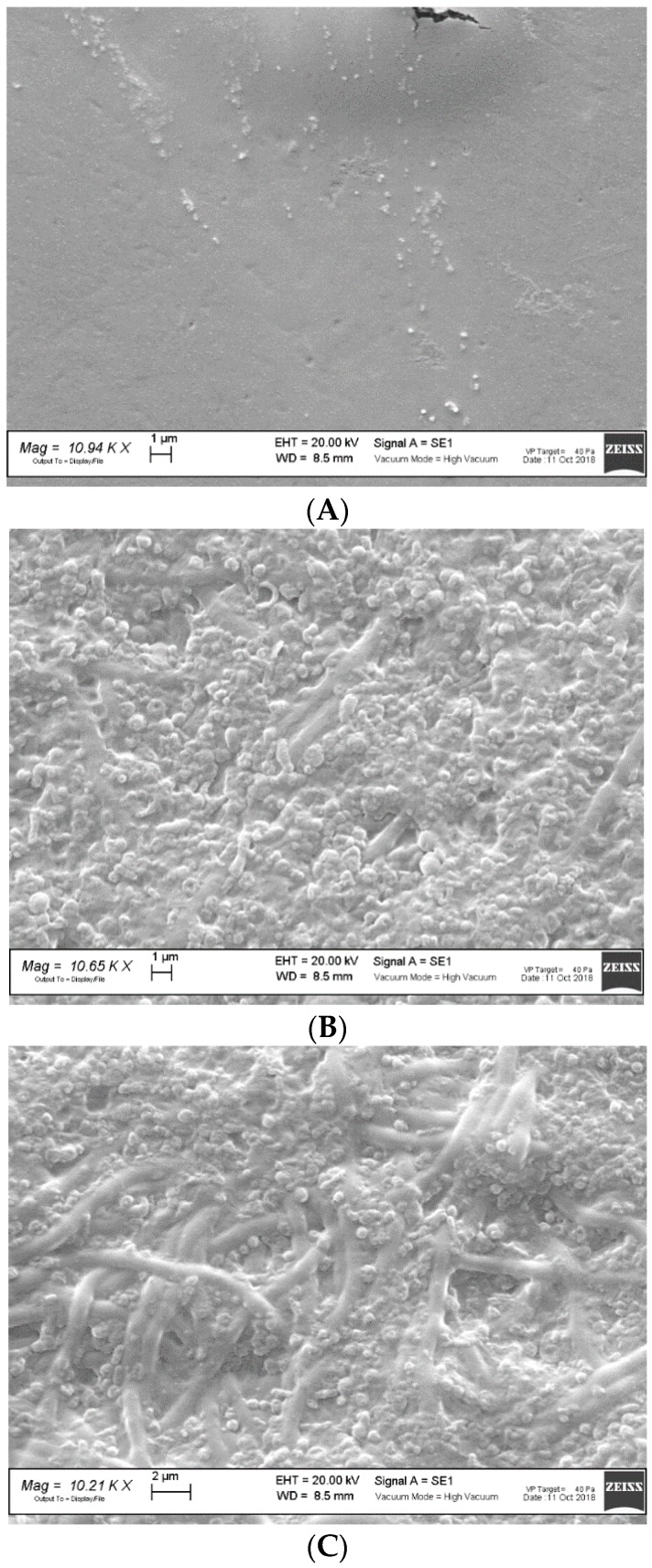
SEM microphotographs of unloaded scaffolds (**A**) CH; (**B**) CH/CS; and (**C**) CH/HA subjected to lysozyme for 10 days.

Figure 2 reports the SEM microphotographs of unloaded scaffolds (A) CH; (B) CH/CS; and (C) CH/HA subjected to lysozyme for 10 days. 

Nanofibrous structure of CH scaffold was completely lost after 10 days of contact with lysozyme. While CH/CS scaffold and mainly CH/HA scaffold were characterized by the presence of some nanofibers submerged in a non-structured material and this could be due to a higher resistance towards the degradation, conceivably to the interaction of chitosan aminogroups (positively charged) with either sulfate groups of chondroitin sulfate or carboxylic ones of hyaluronic acid (both negatively charged). Such an interaction could prevent the loss of the system morphology, although the chitosan enzymatic degradation occurred.

### 3.2. AgNP Nanofibrous Scaffolds Chemico-Physical Characterization

**Figure 3:**
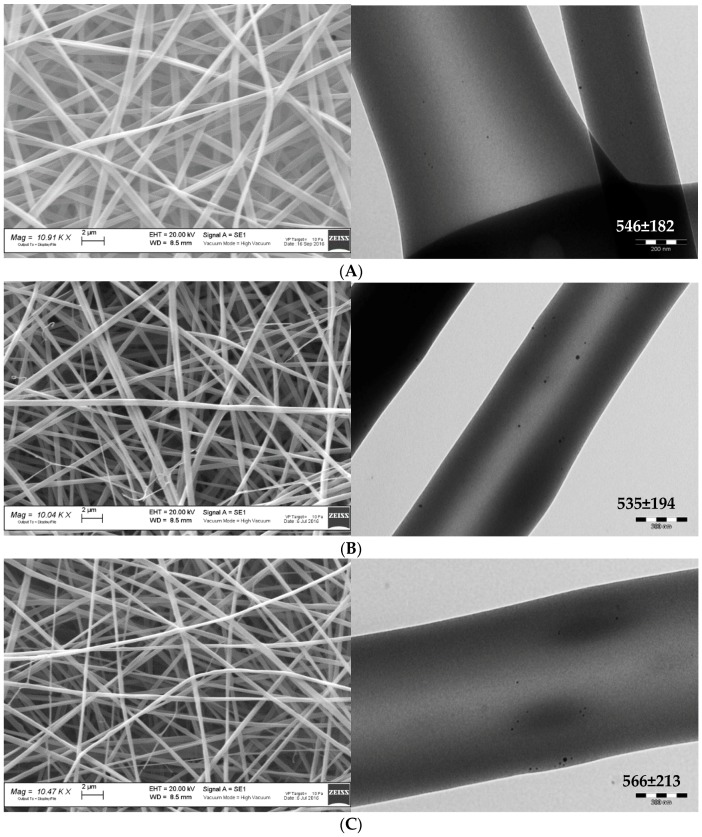
SEM (left column) and TEM (right column) microphotographs of nanofibrous scaffolds loaded with AgNPs: (**A**) CH; (**B**) CH/CS; and (**C**) CH/HA. The mean diameters of nanofibers are reported in TEM microphotographs.

Figure 3 reports SEM (left column) and TEM (right column) microphotographs of nanofibrous scaffolds loaded with AgNPs: A) CH; B) CH/CS; and C) CH/HA. In TEM microphotographs, the mean diameters of nanofibers are reported.

SEM analysis evidences that the nanofibers were continuous and randomly oriented, furthermore they were uniform with smooth surface. Only few defects could be detected when glycosaminoglycans were present. However, both CS and HA did not influence the fiber dimensions and all the scaffolds were characterized by diameters close to 500 nm for all the compositions. The presence of AgNPs was highlighted using TEM. In all the scaffolds, the AgNPs could be visualized as black spots, being much more electrondense than the polysaccharidic matrix that were dipped in. This supports that AgNPs were stable during the preparation of polymer blends and the electrospinning process.

**Figure 4:**
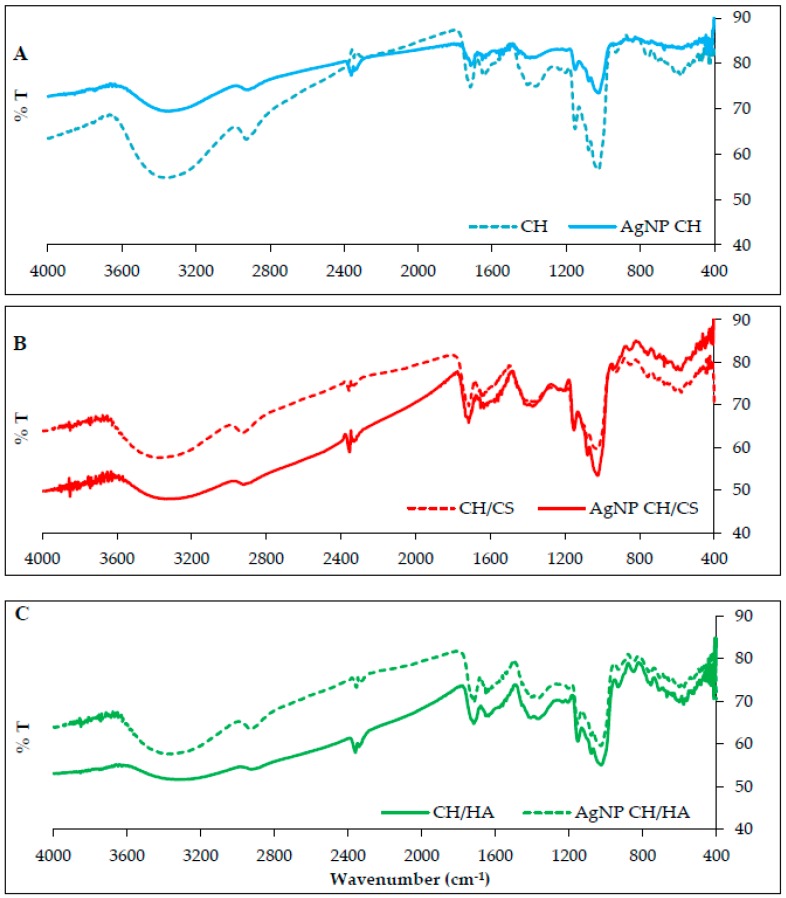
FTIR of nanofibrous scaffolds loaded with AgNPs compared to unloaded scaffolds: (**A**) CH; (**B**) CH/CS; and (**C**) CH/HA.

Figure 4 reports the FTIR spectra for unloaded and AgNPs loaded scaffolds. As for CH scaffold, in the region around 1640 cm^−1^ there was the typical signal of the Amide I band, conceivably related to the occurrence of covalent bonds between chitosan aminogroups and carboxylic moieties of citric acid in accordance with a previous investigation [9]. As for both the CH/CS and CH/HA scaffolds, the peak at 1640 cm^−1^ was less marked and this probably suggested that glycosaminoglycans either chondroitin sulfate or hyaluronic acid could prevent the formation covalent bonds between CH and CA. The scaffold loading with AgNPs did not evidence the formation of new chemical bonds [9]. The substantial identity of the FTIR spectra obtained for AgNPs loaded and unloaded scaffolds was largely expected, due to the low content of AgNPs in the scaffolds, as also proved by TEM images, and due to the negligible number of functional groups of the polymeric matrix surrounding AgNPs, which could interact with the metallic surface of the nanoparticles.

### 3.3. In Vitro Cells Adhesion and Proliferation

**Figure 5:**
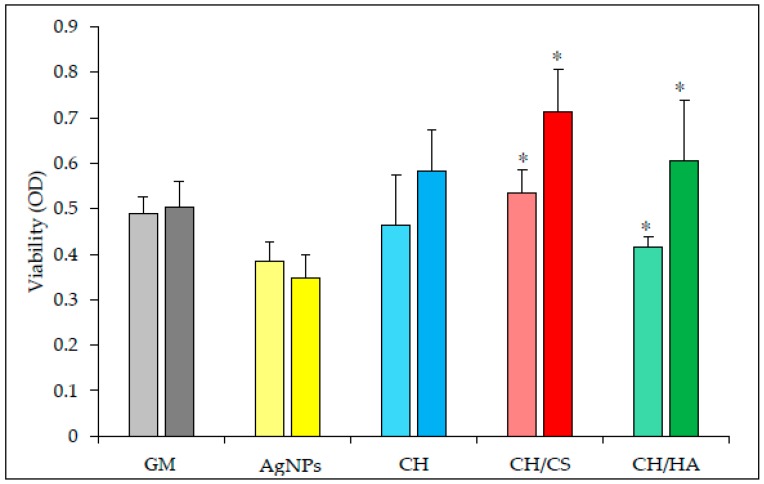
Viability (optical density OD) of fibroblasts in contact with AgNPs as suspension, and grown onto electrospun AgNPs loaded scaffolds, CH, CH/CS, and CH/HA (all containing the same amounts of AgNPs), in comparison to the positive control GM (growth medium, as standard growth conditions) (left bar 3 days/right bar 7 days) (mean values ± sd; n = 8). Statistics: multiple range test: p < 0.05: 3 days: AgNPs vs. CH/CS; 7 days: AgNPs vs. CH; AgNPs vs. CH/CS; AgNPs vs. CH/HA; AgNPs vs. GM; CH vs. CH/CS; CH/CS vs. GM; 3 days vs. 7 days: CH/CS and CH/HA (* significantly different values).

Figure 5 reports the viability (optical density, OD) of the fibroblasts grown onto the AgNPs loaded scaffolds in comparison to AgNPs colloidal suspension (AgNPs concentration as those contained in scaffolds). After 3 days of growth, all the scaffolds determined cell viability to not be significantly different with respect to GM (growth medium, as control) and to AgNPs, as colloidal suspensions, except for the CH/CS scaffold. This significantly supported fibroblast growth with respect to AgNPs, as colloidal suspension, thus suggesting that the CH/CS scaffold possessed a protective effect towards the silver cytotoxic effect.

After 6 days, the differences in performance of the scaffolds were more evident: all the scaffolds were characterized by a significantly better performance in fibroblast growth than AgNPs, as colloidal suspension, confirming that AgNPs scaffolds were able to decrease the toxic effect of silver. Moreover, CH/CS and CH scaffolds were able to improve cell proliferation with fibroblast growth significantly higher than that of the control (GM). Moreover, the scaffolds containing glycosaminoglycans, either chondroitin sulfate or hyaluronic acid, were able to support fibroblast proliferation with a cell growth after 7 days significantly higher than that after 3 days.

**Figure 6:**
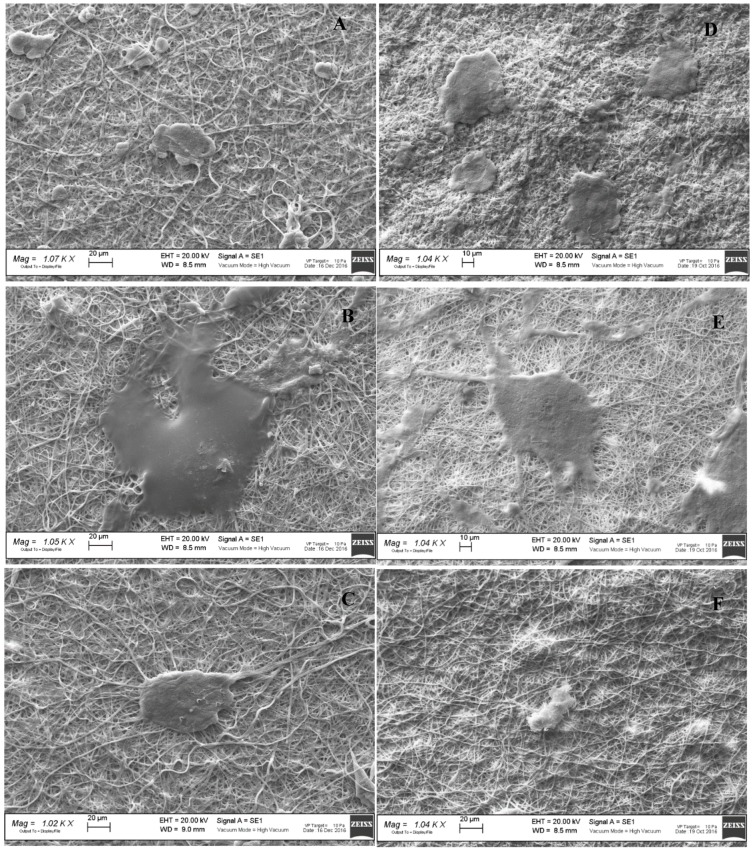
SEM microphotographs of fibroblasts onto electrospun AgNPs loaded scaffolds, (**A**) CH; (**B**) CH/CS; (**C**) CH/HA after 3 days of growth and (**D**) CH; (**E**) CH/CS; and (**F**) CH/HA after 6 days of growth.

**Figure 7:**
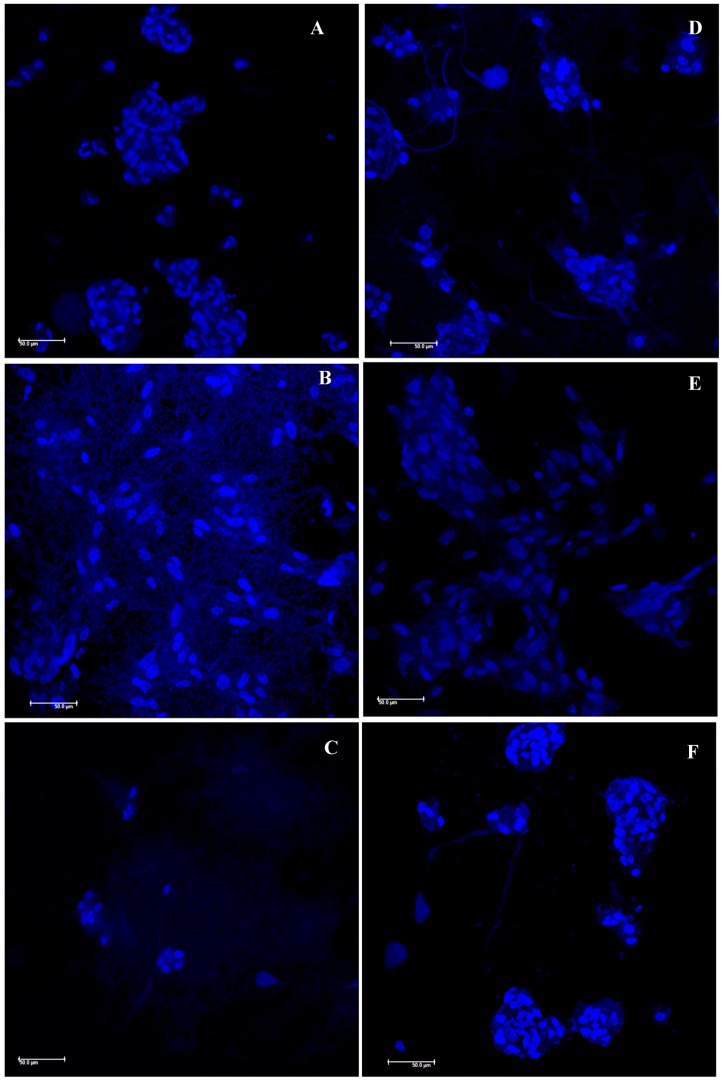
CLSM microphotographs of fibroblasts onto electrospun AgNPs loaded scaffolds, (**A**) CH; (**B**) CH/CS; (**C**) CH/HA after 3 days of growth (left column) and (**D**) CH; (**E**) CH/CS; (**F**) CH/HA after 6 days of growth (right column), (in blue nuclei).

Figure 6 reports the SEM microphotographs of fibroblasts grown onto the AgNPs loaded scaffolds after 3 (left column) and 7 (right column) days. SEM analysis confirms viability data. Furthermore, the images suggest that when fibroblasts grew onto CH/CS scaffold they were nicely attached onto the scaffold nanofibers and spread all over the surface reaching the subconfluence. On the contrary CH and CH/HA scaffolds allowed the fibroblasts adhesion and growth but cell morphology presented a cluster like behavior probably due to an aggregation of cells, thus having a less evident spreading all over the scaffolds. This behavior was further confirmed by CLSM images (Figure 7 left column 3 days of growth and right column 7 days of growth): fibroblasts (relative positions localized by means of cell nuclei) grown onto CH and CH/HA scaffolds were close to each other confirming the hypothesis argued from SEM analysis, while fibroblasts (relative positions localized by means of cell nuclei) grown onto CH/CS scaffold were spread all over the scaffold, and this could be related to the typical behavior of the normal stretched fibroblasts.

### 3.4. In Vitro Antimicrobial Properties

**Figure 8:**
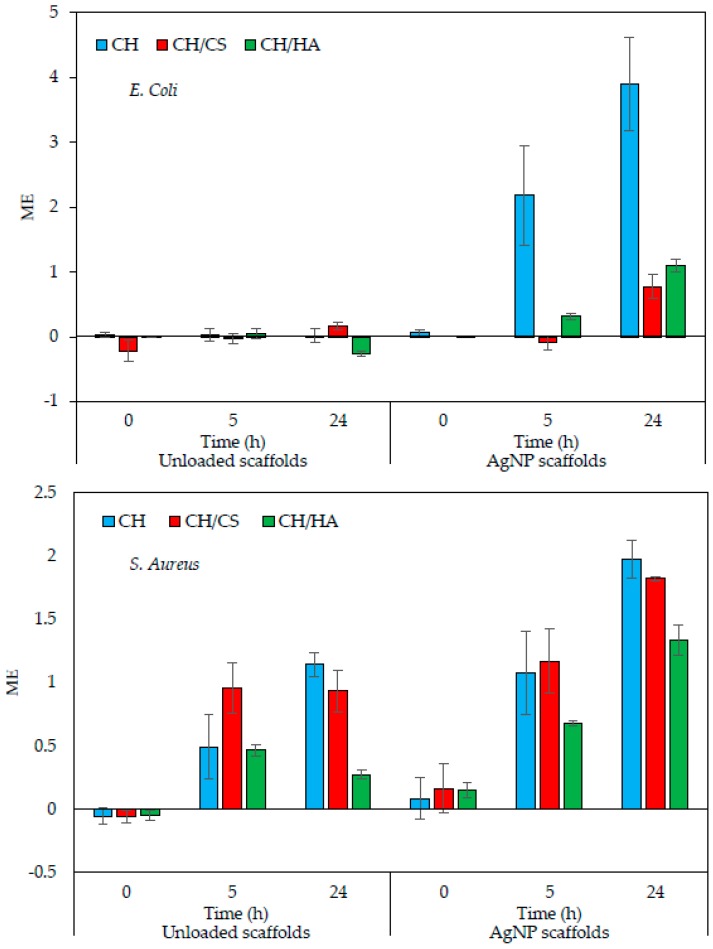
ME (microbicidal effect) against *Escherichia coli* and *Staphylococcus aureus*, evaluated for AgNPs loaded nanofibrous scaffolds (CH, CH/CS, and CH/HA) in comparison to unloaded scaffolds (mean values ± sd; *n* = 3).

Figure 8 reports the results (ME—microbiocidal effect) of antimicrobial properties evaluated against *S. aureus* and *E. coli*, Gram + and Gram—microorganisms, respectively. Unloaded and AgNPs loaded scaffolds were compared. 

The skin microbiota and microenvironment influence the wound repair process and the occurrence of skin infections. Skin wounds offer an environment suitable for microorganism proliferation and when the healing is delayed, wound normal microbiota is replaced by aggressive microbial types. Chronic wounds, which are the more critical one, are firstly colonized by Gram + bacteria and *Staphylococcus aureus* appears the most. In advanced phases, Gram - bacteria (including also *Escherichia coli*) are mostly present and are likely to enter the deeper skin layers, significantly affecting tissues [19]. For these reasons, antimicrobial properties against *E. coli* and *S. Aureus* were assessed.

When *E. coli* was considered, the unloaded scaffolds were not able to cause a reduction of bacteria, indicating a lack of antimicrobial properties against the strain evaluated, over time up to 24 h. This could be due to the partial formation of covalent bonds between the chitosan aminogroups and carboxylic moieties of citric acid. In fact, in a previous work, the chitosan activity against *E. coli* was referred as mediated by electrostatic forces between microbial cell surface and the chitosan aminogroups [20]. 

AgNPs loading conferred to the antimicrobial properties of all the scaffolds with an increasing effect over time. CH scaffold showed the best performance, and this could be attributable to chitosan, although this activity was not present when glycosaminoglycans were the components of the scaffolds. This behavior was probably due to the chitosan GAG interactions that could shield chitosan positive charges mainly responsible for its antimicrobial properties.

As for *S. aureus*, the unloaded scaffolds were characterized by antimicrobial activity up to 24 h with an onset after 5 h of contact. The AgNPs loading of the scaffolds increased their antimicrobial activity that could be detected as soon as the scaffolds were in contact with the bacteria (0 h). The antimicrobial activity increased over time up to 24 h.

Generally, AgNPs scaffolds were characterized by higher antimicrobial properties against Gram + rather than against Gram -. The association of chitosan to a glycosaminoglycan seemed to reduce antimicrobial properties although CH/CS retained them. This is an important finding since the scaffolds were a good substrate to support fibroblasts adhesion and proliferation together with antimicrobial properties.

## 4. Conclusions

Glycosaminoglycan scaffolds based on pullulan, chitosan and chondroitin sulfate or hyaluronic acid were prepared by means of electrospinning with a simple and one step process, starting from polymeric blends in water and acetic acid mixture. The scaffolds were crosslinked by the heating process to obtain water resistant systems and this could also be considered as a sterilizing process. Subsequently their enzymatic degradability was assessed in vitro using lysozyme, normally secreted by macrophages and polymorphonuclear neutrophilis during the inflammatory phase of the healing process. This was a preliminary demonstration to state the suitability of the scaffolds to be used as dermal substitutes. Subsequently the scaffolds were loaded with silver nanoparticles. 

AgNP scaffolds were characterized by nanofibers having smooth surface, regular and uniform shape and AgNPs embedded into the polymeric matrix forming the fibers.

All the scaffolds developed (containing chondroitin sulfate or hyaluronic acid or without glycosaminoglycan as reference) were characterized by the capability to allow fibroblast adhesion and growth onto the systems up to six days and AgNPs entrapped in the scaffolds did not impair cell proliferation, highlighting the capability of these systems to protect the cells from the cytotoxic actions of silver, especially higher when in nanoparticulate form. These systems were also characterized by antimicrobial activity mainly against *Staphylococcus aureus*. 

Finally, the scaffold based on chondroitin sulfate in association with chitosan and pullulan was characterized by the best performance and seems to be a promising tool to treat chronic skin lesions prone to infections as venous leg ulcers, diabetic foot, bed sores and also burns or surgical lesions difficult to heal in elder people.

## 5. Patents

Sandri, G., Bonferoni, M.C., Rossi, S., Ferrari, F., Electrospun nanofibers and membranes, PCT/IT2017/000160, 2017.

## Figures and Tables

**Table 1:** Composition (% *w*/*w*) of polymeric blends used to be electrospun.

						AgNPs Loaded	Unloaded
% *w*/*w*	P	CH	CA	CS	HA	AgNPs/Acetic acid	Water/Acetic acid
CH	10	2.5	2.5	--	--	55/45	55/45
CH/CS	10	2.5	2.5	0.5	--	55/45	55/45
CH/HA	10	2.5	2.5	--	0.5	55/45	55/45
